# The SUMMIT ambulatory-ICU primary care model for medically and socially complex patients in an urban federally qualified health center: study design and rationale

**DOI:** 10.1186/s13722-018-0128-y

**Published:** 2018-12-14

**Authors:** Brian Chan, Samuel T. Edwards, Meg Devoe, Richard Gil, Matthew Mitchell, Honora Englander, Christina Nicolaidis, Devan Kansagara, Somnath Saha, P. Todd Korthuis

**Affiliations:** 1 Division of General Internal Medicine and Geriatrics, Oregon Health and Science University, 3181 SW Sam Jackson Park Road L475, Portland, OR 97239-3098 USA; 2 Central City Concern, Portland, OR USA; 3 Portland VA Medical Center, Portland, OR USA; 4 Division of Hospital Medicine, Oregon Health and Science University, Portland, OR USA; 5 School of Social Work, Portland State University, Portland, OR USA

**Keywords:** Primary care innovation, Health service delivery, Patient experience, Patient centered medical home, Partnered-research, Complex care, Homelessness, Substance use

## Abstract

**Background:**

Medically complex urban patients experiencing homelessness comprise a disproportionate number of high-cost, high-need patients. There are few studies of interventions to improve care for these populations; their social complexity makes them difficult to study and requires clinical and research collaboration. We present a protocol for a trial of the streamlined unified meaningfully managed interdisciplinary team (SUMMIT) team, an ambulatory ICU (A-ICU) intervention to improve utilization and patient experience that uses control populations to address limitations of prior research.

**Methods/design:**

Participants are patients at a Federally Qualified Health Center in Portland, Oregon that serves patients experiencing homelessness or who have substance use disorders. Participants meet at least one of the following criteria: > 1 hospitalization over past 6 months; at least one medical co-morbidity including uncontrolled diabetes, heart failure, chronic obstructive pulmonary disease, liver disease, soft-tissue infection; and 1 mental health diagnosis or substance use disorder. We exclude patients if they have < 6 months to live, have cognitive impairment preventing consent, or are non-English speaking. Following consent and baseline assessment, we randomize participants to immediate SUMMIT intervention or wait-list control group. Participants receiving the SUMMIT intervention transfer care to a clinic-based team of physician, complex care nurse, care coordinator, social worker, and pharmacist with reduced panel size and flexible scheduling with emphasis on motivational interviewing, patient goal setting and advanced care planning. Wait-listed participants continue usual care plus engagement with community health worker intervention for 6 months prior to joining SUMMIT. The primary outcome is hospital utilization at 6 months; secondary outcomes include emergency department utilization, patient activation, and patient experience measures. We follow participants for 12 months after intervention initiation.

**Discussion:**

The SUMMIT A-ICU is an intensive primary care intervention for high-utilizers impacted by homelessness. Use of a wait-list control design balances community and staff stakeholder needs, who felt all participants should have access to the intervention, while addressing research needs to include control populations. Design limitations include prolonged follow-up period that increases risk for attrition, and conflict between practice and research; including partner stakeholders and embedded researchers familiar with the population in study planning can mitigate these barriers.

*Trial registration* ClinicalTrials.gov NCT03224858, Registered 7/21/17 retrospectively registered https://clinicaltrials.gov/ct2/show/NCT03224858

## Introduction

A small group of high cost-high needs patients (HCHN) accounts for a disproportionate percentage of health care expenditures [1, 2]. These patients often have multiple medical and psychiatric comorbidities and functional impairments [3] that lead to costly and unnecessary care [4], and have increased risk for adverse drug events [5]. The number of specialty physicians involved in their care also increases risk of fragmentation of care [6], and poor transitions of care from hospital to home [7–9]. In addition, highly prevalent adverse social factors, such as poverty, homelessness, and substance use disorders increase the risk for overuse of hospital and emergency departments (ED), as well as underuse of primary care [10].

As healthcare systems and Accountable Care Organizations (ACOs) assume more financial risk for quality of care delivered to patients, there are efforts to focus and intensify resources for HCHN patients [11]. Innovative intensive primary care (IPC) programs employ a variety of approaches to improve care quality and reduce utilization, including use of multi-disciplinary care teams, increased primary care access, improved coordination and continuity of primary care, and enhanced self-efficacy through counseling or linkages to social services/case management [2, 12]. However, there is unclear evidence for IPC effectiveness. A recent systematic review of IPCs showed mixed effects on utilization [13]. The only study targeting patients with complex social needs examined the VA homeless patient aligned care team (H-PACT) program, an intensive “ambulatory-ICU” (A-ICU) program for homeless Veterans who were unwilling or unable to access traditional primary care [14]. While 6-months pre- and post-enrollment analyses showed a 25% reduction in combined hospitalization and ED utilization, the evaluation lacked a control group. Other gaps in the literature include few interventions targeting HCHN patients in urban community health centers, and those with substance use disorders and co-occurring severe mental illness, independent risk factors for utilization of healthcare services [15–17].

There are several reasons for this gap. These patients are difficult to recruit in studies because of their social complexity, and distrust of medical system and may require partnered collaboration between researchers and community-based clinics beyond traditional research methods [18]. Furthermore, these programs occur in real-time in response to stakeholder and patient needs, and evaluation plans are often lower priority than service delivery—there are few learning health systems that have the resources or expertise to conduct evaluation activities in Federally Qualified Health Center (FQHC) populations [19, 20].

We designed SUMMIT (Streamlined Unified Meaningfully Managed Interdisciplinary Team) to address some of these gaps. We describe a practiced-based research partnership between Old Town Clinic (OTC), an FQHC, and Oregon Health & Science University (OHSU), a research institution, in design of a randomized, wait-list control trial to assess whether an A-ICU model of care compared to existing patient centered medical home (PCMH) care improves healthcare utilization, patient experience, and self-efficacy at 6 months for medically and socially complex patients in an urban healthcare for the homeless setting.

## Methods/design

### Study design and rationale

**Fig. 1:**
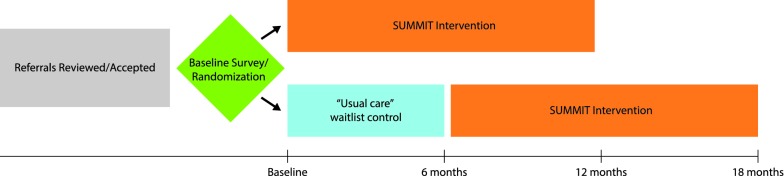
Study timeline for SUMMIT study using “wait-list control” design. Participants are randomized to start immediately in SUMMIT or continue “usual care” for 6 months before joining SUMMIT

SUMMIT is a randomized controlled study using a wait-list control design (see Fig. 1), described in prior practice-based research literature [21, 22]. Patients are randomized to start the SUMMIT intervention immediately, or continue with usual primary care for 6 months before crossing over to the SUMMIT intervention with data collection at multiple time-points. Thus, we address limitations of other practiced-based evaluations of IPC interventions that use pre-post designs without control groups. Furthermore, compared to a randomized controlled trial, the wait-list control design is more acceptable to clinic staff, patients, and payer stakeholders who may consider it unethical to deny patients access to the intervention. The wait-list control design permits gradual ramp-up of staffing.

### Study setting

We recruit study participants from the OTC, an integrated medical and behavioral healthcare clinic and FQHC in Portland, Oregon. OTC serves over 5000 patients who receive primary care services at the clinic through four PCMH teams. The average age of OTC patients is 48.7 years (± 12.5), and a majority are male (60.9%), White/Non-Latino race/ethnicity (72.2%) with very low incomes (46.5%); about one-third (30%) have at least two chronic medical conditions. Many of OTC patients are referred from housing programs or substance-use treatment programs—67% of OTC patients in 2017 were homeless or unstably housed, and 61% of patients had a substance use disorder diagnosis. In addition to primary care, the clinic provides co-located mental health services delivered by psychiatric nurse practitioners; substance use counseling services and pharmacotherapy for alcohol and opioid use disorders; chronic disease pharmacists and an on-site pharmacy and lab services; wellness and activity classes, including daily acupuncture; integrated occupational and physical therapy, and social work services. Additionally, teams have access to community health workers to assist with patient engagement with primary care. Despite this specialized care, a subset of OTC patients continued to consume provider and staff attention and over utilize hospitals and EDs. A 2014 needs assessment showed 25% of OTC’s patients had at least one hospitalization or six ED visits in the preceding year, and an additional 14% had 2–5 ED visits in the preceding year, spurring development of the SUMMIT intervention.

Starting in 2006, OHSU and OTC initiated a clinical partnership with the goal of working together to identify research opportunities in healthcare disparities, integrated care models and improved access for underserved populations. OHSU-affiliated providers staff OTC clinics, supervise resident continuity clinics, and developed a social medicine curriculum for medical students. More recently, OHSU clinician-researchers embedded at OTC have leveraged the partnership with OTC to design clinical innovations on hospital-based transitions of care, and substance use treatment that utilizes the strengths of each organization [23, 24]. These prior studies were successful in part due to intimate knowledge of the culture of the clinic, patient populations needs, and an awareness of the needs of the partner. Interest in a novel A-ICU care model prompted opportunity to utilize this research collaboration.

### Eligibility criteria and recruitment

Box 1 summarizes participant inclusion and exclusion criteria. Primary care providers (PCPs) are encouraged to refer patients whose medical complexity, combined with social and behavioral factors, made it difficult for existing care teams to deliver optimal care. PCPs complete a referral form for patients that meet medical burden criteria (at least one medical condition including congestive heart failure, uncontrolled diabetes, advanced COPD [World Health Organization group C or D], chronic kidney disease (≥ stage 3), end-stage liver disease, chronic or severe soft tissue infections, osteomyelitis, or failure to thrive), utilization criteria (≥ 1 hospitalization or ED visit in the prior 6 months), and substance use disorder defined by referring PCP or chart diagnosis, or behavioral health criteria (≥ 1 psychotic disorder, mood disorder, post-traumatic stress disorder [PTSD]).

**Box 1:** Summit A-ICU study enrollment criteria

Enrollment criteria
1 or more medical/surgical hospitalizations in last 6 months
1 or more of the following medical conditions:
Chronic kidney disease stage III
Congestive heart failure
Chronic obstructive pulmonary disease, group C/D
Diabetes with A1c > 8%
End Stage Liver Disease (ESLD)
Osteomyelitis/severe soft-tissue infection
Or 1 or more of the following co-morbid conditions
Mental health
Substance use disorder
Difficulty engaging in usual primary care (missed appointments)
Exclusion criteria
Inability to consent (as demonstrated by “teach-back” method)
Non-English speaking patients
On hospice or deemed < 6 months to live at time of enrollment
Diagnosis of terminal cancer
Inability to participate in follow up assessment due to aphasia, severe hearing impairment or behavioral issues.

The SUMMIT team reviews referrals at regular intervals to confirm clinical appropriateness. Following acceptance to the team, the research assistant (RA) approaches the patient to determine study eligibility, obtain consent and complete the baseline survey. We exclude potential participants if they are non-English speaking, are on hospice at time of enrollment (< 6 months to live), diagnosed with terminal cancer diagnosis, are unable to consent using “teachback” method [25], or are unable to follow-up by phone due to aphasia, and/or severe hearing impairment at time of enrollment.

### Participant sample recruitment procedures and timeline

Once consent is obtained, the RA administers the baseline survey that includes demographic, bio-psychosocial assessments and baseline assessment of patient-reported outcomes (Fig. 1). Following completion of the baseline survey, the participant is randomized to immediate entrance into the SUMMIT intervention or placement on a 6-months waitlist. Study participants receive $5 gift cards for each completed survey.

If the participant is randomized to the wait-list control group, they remain in usual care for 6 months. After 6 months, the RA contacts the participant to repeat the study assessments, and the participant transfers care to the SUMMIT team. If the participant is randomized to the intervention group, they are scheduled for an initial intake appointment with the SUMMIT team. The RA contacts participants at 6 and 12 months from date of enrollment for ascertainment of outcomes with a window of 6 weeks defined as 2 weeks prior and 4 weeks after the due date. Participants are followed for 12 months after SUMMIT enrollment (up to 18 months if assignment to wait-list control group).

We sought strategies to improve engagement with participants and minimize loss to follow up, given the complexity of the target population. We asked consented participants to list additional phone numbers and/or addresses as points of contact as part of baseline survey procedures. We convened a patient advisory group, and included in our research meetings a patient liason to introduce the SUMMIT study to obtain feedback from patient advocates regarding study recruitment and follow-up procedures.

### Study integrity and randomization

The study design conforms to CONSORT statement recommendations for randomized clinical trials of non-pharmacologic treatment [26]. Upon completion of consent and baseline survey, participants are randomized to either immediate start with the SUMMIT team or be placed on a 6-months wait-list with a 1:1 allocation per computer-generated randomization schedule. We used sequentially numbered, opaque, sealed envelopes to attain allocation concealment. A member of the research team not involved in the consent and enrollment process generated the allocation sequence. Following allocation of group assignment, the participant, clinic staff, and RA conducting follow up surveys will not be blinded. The research members conducting the statistical analysis will be blinded from treatment assignment. We will conduct an intent-to-treat analysis.

### Ethics and dissemination

The study protocol was reviewed and approved by the OHSU IRB in 2016 (IRB 15285). The study also received a Certificate of Confidentiality from the National Institutes of Health to protect data on drug and alcohol use. The protocol is registered with clinicaltrials.gov (NCT03224858). None of the investigators have financial or competing interests in conflict with the aims of the trial.

## Study arms

### Intervention: SUMMIT intervention description

The intervention is a clinic team of co-located multi-disciplinary staff with reduced panel size and flexible scheduling (Box 2). Staffing consists of two half-time physicians (1.0 FTE) with board certification in addiction medicine, one complex care nurse, two care coordinators, two licensed clinical social workers (LCSW), pharmacist, team manager, and quality analyst. All team members have additional training in Motivational Interviewing, patient goal setting, and palliative care principles. These didactic trainings were delivered by partner community organizations during weekly dedicated non-clinical time built into the SUMMIT schedule. The aims of the SUMMIT team are closely aligned with Shippe and Montori’s theory of cumulative complexity, emphasizing ways to increase patient self-efficacy and decrease treatment burden [27]. Core activities (Box 3) include an initial comprehensive intake with medical and behavioral team members, patient driven health goal setting, transitional care protocols when patients experience hospitalizations, medication management assessment, weekly panel review, and case management to address social determinants of health and other unmet needs. The team has flexibility to conduct home visits, facilitate unscheduled clinic visits, accompany patients to specialty appointments, and deliver addictions treatment including medications for addiction treatment. The quality analyst assists the team in developing “Plan-Do-Study-Act” cycles and panel management activities.

**Box 2:** Summit A-ICU intervention description and team structure

Team structure
*Care coordinator*—the patient’s primary point of contact, the care coordinator assists with patient follow up, acts as scribe for physician face-to-face visits, conducts outreach activities with the goal of enhancing rapport building
*Team manager*—the team manager coordinates patient and team schedules, interfaces with clinic operations and administration, conducts outreach, and leads team activities, including organizing trainings, and process improvement cycles
*Physician*—General internist with additional board certification in addiction who provides front line care to patients including acute and chronic disease management, advanced care planning, medication management, and coordination of care with specialists
*Social worker*—a licensed clinical social worker embedded in the team who meets with the patients on Day 1 to elicit social vulnerabilities and provide counseling + case management support to patients as needed
*Complex care nurse*—a nurse that provides medical triage services, transitional care planning, and assists patients with health education activities as well as outreach (accompanying patients to specialty appointments)
*Pharmacist*—the pharmacist works with patients and team members to assist with medication reconciliation, transitions of care, and chronic disease medication management for patients (diabetes, heart disease) with the goal of reducing medication treatment burden

**Box 3:** Key features and core activities of summit A-ICU

1. *Transfer of care to the co-located stand-alone team* Patients transfer care from existing primary care to the SUMMIT team to encourage coordinated, unified care from a single team. Co-location offers opportunity to facilitate interdisciplinary meetings, as well as informal conversations to enact care plans during non-visit time
2. *Comprehensive initial intake* The first visit(s) include a 60 min social intake with the Social worker, followed by a 60 min medical intake with provider/care coordinator with open ended questions and focus on patient health goal elicitation and assessing self-efficacy and treatment burden. The visit forms the basis for the patient-centered, goal-based care plan
3*. Interdisciplinary team reviews* Following intake, subsequent activities and appointments are determined based on patient status, medical and psychosocial complexity. Set time aside for daily huddles provide opportunity to discuss the patients scheduled for the day, and recently hospitalized or discharged patients. Weekly “speed dating” rounds provide opportunity to review existing patients assess whether current interventions are working and revise care plans as necessary
4. *Transitions of care coordination/tracking* Led by the complex care nurse, and pharmacist, the SUMMIT team developed protocols for coordination of care for hospitalized patients to communicate pertinent information to inpatient care teams, and develop follow up care plans prior to patient discharge
5*. Built-in counseling services* Led by the social worker but supported by all team members who are trained in motivational interviewing, trauma-informed care, and cognitive behavioral therapy. Social workers provide individualized counseling and leverage existing linkages to mental health prescribers as necessary
6. *Navigation of social services* Team members assist patients with long term care planning, advanced directives, linkages to community resources such as disability, housing benefits
7. *On-demand availability and access to off-hours warm lines* A separate SUMMIT team phone number is available for patients and patient caregiver teams to access SUMMIT team members at all hours of the day. SUMMIT physicians cover the phone during off-clinic hours to respond to patient care needs and concerns
Additional activities/flexibility
8. *Outreach visits* Team members are available to conduct outreach visits for patients on an as needed basis. Visits are used as an opportunity to assess patients outside the clinic, develop rapport and trust, and facilitate and support a health related activity (i.e. Accompaniment to specialist referrals, delivery of medications if temporarily homebound, assistance with access to social services)
9. *Pharmacy education/chronic disease medication management* For select patients with non-controlled chronic disease conditions (diabetes, hypertension, heart disease), the SUMMIT pharmacist is available for 1 to 1 consultation and medication review and management. The pharmacist is empowered to enact the care plan and titration or tapering of medications
10. *In-visit scribing* Care coordinators sit in on medical visits with provider and patients and scribe for the provider. This activity promotes unified communication of the care plan between patient, care coordinator, and physician; and allows improved patient experience during face-to-face visits

### Funding SUMMIT

SUMMIT is funded through reallocation of existing staff resources and clinical operations budget, rather than grants. Funding for on-going operations is through a negotiated per-member per-month payment for participants in the care model and fee-for-service payments for patient visits with a regional Medicaid payor.

### Wait-list controls: treatment as usual

**Fig. 2:**
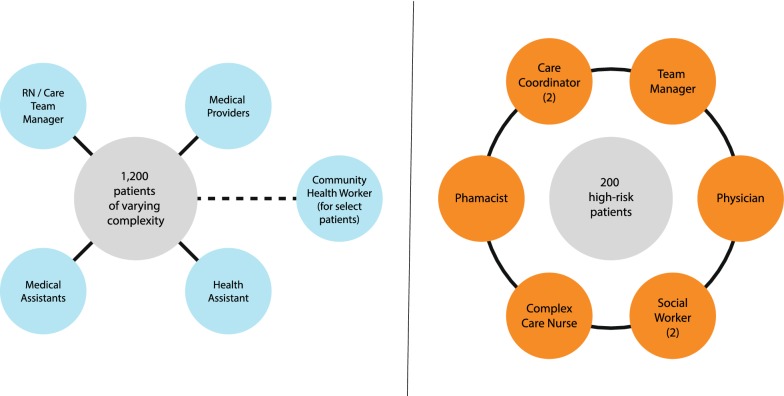
Comparison of “usual care” team (left) and SUMMIT A-ICU (right)

Usual care operates as a PCMH model (see Fig. 2). Within the clinic, there are four teams consisting of PCP, care team manager (usually a licensed practical nurse), medical assistants, and health assistants who handle clerical and phone communication duties. Patients have referral access to on-site non-medical services described above, including addictions treatment, housing referrals, and behavioral health. In 2015, OTC introduced embedded CHW workers in partnership with a regional Medicaid payor [2]. The CHWs have training in addictions or mental health counseling and engage HCHN patients outside the clinic setting. Activities of the CHW include motivational interviewing, case management, advocacy, facilitation of multi-disciplinary care planning, collaboration with primary care, and individual resource building for short-term (< 6 months) engagement.

## Measurement

### Data collection

The RA administers baseline and follow up surveys to participants using paper questionnaires and then enters responses into a REDCap database designed with branching logic, range checks, and forced functions to increase data quality. The RA is independent from the clinical teams to decrease risk of social desirability bias in survey response. The RA stores paper questionnaires in a locked file cabinet on site in case of discrepancies in the REDCap database. We use administrative data to examine care utilization outcomes. If a participant is lost to follow up or does not engage with the care team, we will still be able to obtain inpatient and outpatient utilization data.

## Outcomes

### Primary outcome

The primary outcome is total inpatient hospitalizations at 6 and 12 months after study enrollment as assessed by PreManage, a consortium of local state and cross-state hospitals that sends in real-time hospital administrative event information (inpatient admissions and discharges, ED visits) to health plans and provider groups.

### Secondary outcomes

The secondary outcomes are ED utilization at 6 and 12 months after study enrollment, and patient reported activation at 6 and 12 months of the intervention. We assess patient activation using the Patient Activation Measure (PAM-13) [28, 29], a validated tool used widely in research to assist in tailoring care plans, and evaluation assessment.

### Exploratory outcomes

We will also consider pre-specified, exploratory outcomes (Table 1). We will use PreManage to calculate average length of stay per inpatient hospitalization; mortality (based on chart record review); outpatient clinic visits; and housing status (assessed using a self-reported survey question: “Which of the following best describes your current residence?”) at 6 and 12 months. We assess patient experience of healthcare at 6 and 12 months using the Consumer Assessment Healthcare Professionals (CAHPS); [30] we also assess patient reported quality of life using the SF-12 survey [31], a single item palliative care measure (Edmonton Symptom Assessment System (ESAS) [32], and a six-item life chaos measure by Wong that assessed self-reported ratings of participants’ routines and daily activities [33]. We will assess the impact of SUMMIT on all OTC clinical staff by assessing a two-item burnout measure periodically during implementation [34].

**Table 1:** Participant timeline and assessments (per SPIRIT guidelines) [50]

Timepoint*	Study period				
	Enrollment/allocation	Post-allocation			Close–out
	− *t*_1_ = 0	6 months	12 months	18 months	18 months
Enrollment					
Eligibility screen	X				
Informed consent	X				
Baseline 1	X				
Allocation	X				
Interventions					
Immediate		X	X		
Wait-list			X	X	
Assessments					
Baseline 1 and 2					
“Please choose the number that best describes how you feel?” (0–10) [32]	X	X	X	X	X
PHQ-9 [38]	X				
AUDIT 10 [40]	X				
Self reported alcohol use disorder history	X				
DAST [40]	X				
Self report substance use disorder history	X				
Tobacco use	X				
Self reported falls over 6 months	X				
Telephone interview for cognitive status (TICS14) [51]	X				
Health Literacy 3 item questionnaire [36]	X				
ESSI 7 social support questionnaire [20]	X				
Food security 2 [3, 7]	X				
Income source	X				
Education	X				
Race-ethnicity	X				
Housing status	X	X	X	X	X
Outcomes					
ED visits 6 months prior	X	X	X	X	X
Hospitalizations 6 months prior	X	X	X	X	X
PAM-13 [29]	X	X	X	X	X
CAHPS-10 [52]	X	X	X	X	X
SF-12v2 [31]	X	X	X	X	X
Chaos Scale 6 [33]	X	X	X	X	X
Mortality/death	X	X	X	X	X

*PHQ* patient health questionnaire, *AUDIT* alcohol use disorder identification test, *DAST* drug abuse screening test, *TICS* telephone interview for cognitive status, *ESSI* Enriched social support instrument, *ED* emergency department, *PAM* patient activation measure, *CAHPS* consumer assessment of healthcare providers and systems, *SF-12* short form health survey

### Socio-demographic and other potential covariates

Self-reported socio-demographic variables include age, gender, race/ethnicity, marital status, educational attainment, and annual household income. Part of the motivation for this study is to learn more about these patients and determine how psychosocial factors, of which little are captured in health records or administrative data, play a role in their healthcare experiences. Therefore, we also assessed perceived social support [35], current living situation, self-reported health literacy [36], and food insecurity [37]. We also screen for depression using the patient health questionnaire (PHQ-9) [38], cognitive impairment using telephone interview for cognitive status (TICS) [39], and drug and alcohol use disorders using the drug abuse screening test (DAST) and the alcohol use disorder identification test (AUDIT) [40]. Measures were proposed during planning meetings, and pilot tested prior to finalizing.

### Sample size calculations

We conducted several sample size calculations to estimate the study size population.

We examined the sample size necessary to demonstrate a 40% reduction in our primary outcomes of hospital utilization over 6 months. We determined average number of hospitalizations over the prior 6 months for a pilot sample of SUMMIT patients (2.3 hospitalizations/person over 6 months, SD = 1.9). Assuming 80% power and 2-tailed alpha, the sample size estimated was 140 participants. Based on preliminary data, we believed 400 existing patients met SUMMIT eligibility for utilization, but targeted an enrollment of 200 based on potential difficulties with recruitment and retention. We used prior literature to determine an effect size of a four-point increase on the secondary outcome of PAM score, with standard deviation of 10; therefore, at 80% power and 2 tailed alpha, the sample size estimated was 196 [41].

### Statistical analyses

We will use descriptive statistics to describe the study population and assess similarity of baseline characteristics between study arms using Chi square, *t*-tests, and Fisher’s exact test. For each outcome, we will conduct intent-to-treat analyses using difference-in-differences regression to compare the study arms during baseline and 6-months and 12-months follow up [42]. Use of a wait-list control design permits two methodologies to assess outcome changes. The first analysis will be a comparison of intervention and control group outcomes at 6-months, adjusting for any baseline covariates that differ by chance in each group. The second analysis will use the wait-list control to conduct a repeated measures analysis controlling for calendar time to account for secular changes in the intervention and enhanced usual care groups over time. We also plan to conduct pre-specified sub-group analyses to identify which SUMMIT patients benefit most from the intervention as currently constructed, including: study participants with only medical complexity; participants with active/primary substance use disorders; and participants who have housing instability at enrollment. Though these subgroup analyses will likely be underpowered, they may inform refined referral criteria or assist in a refined intervention.

### Qualitative and formative evaluation

In addition to the primary evaluation, we are conducting qualitative interviews with clinical staff members and patient participants to explore how the intervention evolves over time and gain insights for what intervention activities worked well and what can be improved. We are conducting formative evaluation through quantitative and qualitative methods to describe intervention components, assess fidelity, and develop lessons learned [43]. This includes tracking number of visits to medical, mental health, pharmacy, nursing providers, tracking whether patients received SUMMIT core activities, and documenting issues affecting implementation of the program as intended.

### Data monitoring

The research team will produce administrative reports on a quarterly basis that describe study progress including: accrual, demographic, study subjects status, outstanding REDCap study forms, error rate pertaining to adherence to inclusion/exclusion criteria and the study protocol. These reports will be reviewed internally for ongoing quality control and submitted at the request of the IRB.

## Discussion

This paper describes a partnered approach to design and evaluation of a novel intensive A-ICU model of primary care for medically and socially complex patients at an FQHC clinic primarily serving low-income patients experiencing homelessness or substance use disorders. The results of this study will contribute to an evolving literature on intensive primary care interventions that addresses two gaps: (1) a need for more practiced-based research studies that include control populations; and (2) a focus on HCHN patients with high rates of homelessness and substance use.

While there is interest in improving quality of care and lowering costs for HCHN patients, how to achieve this is unclear. Intensive primary care interventions are an approach popularized by Camden Coalition’s “Hotspotters” [44] and others like it; however, there are few published evaluations to support investing resources into these models. There are several reasons why these studies do not get published. Many of these intervention programs lack resources or expertise to conduct formal evaluations and dissemination activities—often, programs are implemented, and community partners move on to the next pressing need. Another reason is that multi-component interventions like those for HCHN patients are difficult to describe and vary depending on local context [13, 45]. Patients affected by poverty, homelessness, and substance use are understudied, and have stigma associated with research participation that makes traditional clinical trial participation challenging. Our study protocol addresses these limitations by using a community-partnered approach and embedded researchers that meets both programmatic and research needs.

The wait-list randomized control design of the SUMMIT evaluation offers a balance between research and practice priorities. From a research perspective, incorporating randomization to a control group allows the study to address potential bias resulting from regression to the mean, and minimizes confounding that is present in pre-post designs. From a programmatic perspective, the wait-list control design meets the needs of clinical priorities in that all participants who are accepted have the opportunity to receive the intervention, and allow for gradual scaling up of the intervention over time to meet staff capacity [46, 47]. If the trial is successful, this study design may serve as a model for future evaluations of multi-component, interdisciplinary, practice-based evaluations of interventions for HCHN populations in other settings.

Use of this design is not without trade-offs, including accounting for extended follow-up time that may increase risk for attrition [22]. We limited the wait-list to 6 months and ask for multiple sources of contact information from participants at baseline to help decrease this risk; however, assessing primary and secondary outcomes at 6 months may be too short a time period to detect noticeable differences in utilization and self-efficacy because behavior change interventions often take up-front investments and require extended time horizons (i.e. multiple years) to demonstrate efficacy [48, 49]. Implementing practice-based research often leads to conflicts between research and practice, and our experience has been no exception; however, use of embedded researchers familiar with the patient population and clinic culture, and incorporating input from stakeholders was beneficial for evaluation planning.

In conclusion, we are testing a novel model of primary care using multi-disciplinary teams with reduced panel size and increased flexibility as an intervention to improve quality of care for patients with multiple chronic medical and social complexity. The outcomes of the SUMMIT study will provide real-world evidence about the efficacy of an A-ICU model of care for HCHN patients particularly sensitive to social determinants of health.
